# Exploring the venous supply of the face: An illustrated overview of contemporary literature

**DOI:** 10.1016/j.jpra.2024.10.019

**Published:** 2024-11-06

**Authors:** Jeremy Isaac, Lee Walker, Stephen R. Ali, Iain S. Whitaker

**Affiliations:** a Wish Skin Clinic, Port Talbot, United Kingdom; b B City Clinic, Liverpool, United Kingdom; c Reconstructive Surgery and Regenerative Medicine Research Centre, Institute of Life Sciences, Swansea University Medical School, Swansea, United Kingdom; d Welsh Centre for Burns and Plastic Surgery, Morriston Hospital, Swansea, United Kingdom

**Keywords:** Facial venous anatomy, Facial vein, Venous supply, Facial vasculature

## Abstract

Understanding the vascular anatomy of the face is crucial for ensuring safe clinical practices, especially as aesthetic procedures involving hyaluronic acid fillers are gaining popularity. Although vascular complications from these procedures are rare, there has been a documented increase in adverse events linked to venous and arterial occlusions. This review addresses the knowledge gap regarding the facial venous system compared to the well-documented facial artery system, emphasising the importance of thorough anatomical knowledge to mitigate risks during injectable cosmetic procedures. The complex and variable anatomy of the facial veins, including connections to the intracerebral venous system, allows for the retrograde spread of infections and fillers, with key tributaries such as the angular and infraorbital veins facilitating communication with the cavernous sinus. Notably, the absence of valves in certain venous regions can lead to retrograde filler flow, exacerbating complications. This illustrated review provides an analysis of the facial venous anatomy, focusing on the distribution and depth of venous structures, and aims to equip practitioners with insights that can help reduce the incidence of vascular complications associated with cosmetic procedures.

## Introduction

The vascular anatomy of the face plays a pivotal role in ensuring safe clinical practice, especially as aesthetic procedures such as hyaluronic acid (HA) soft tissue fillers continue to gain popularity. A profound understanding of this anatomy is crucial for mitigating risks associated with these increasingly common procedures. Vascular adverse events, though rare, have been well documented in the literature and are unfortunately on the rise.^1^ Notably, several studies have linked adverse events to a combination of venous and arterial occlusions.^2^ Although the course and variation of the facial artery and its tributaries are well-established, significantly less data exist regarding the facial vein.^3^ This knowledge gap is concerning, given that the venous anatomy of the face can play an essential role in the severity and management of vascular complications during cosmetic procedures.

HA fillers are favoured for their non-invasive, reversible nature and proven safety profile.^4^ However, their expanding use inherently elevates the risk of complications, emphasising the importance of detailed anatomical knowledge, particularly of the facial vasculature.^5^ The literature includes several studies describing adverse events that involve venous and arterial intravascular injections, resulting in severe outcomes such as combined venous and arterial occlusion.^2^^,^^6^ For instance, one study highlighted the role of the ophthalmic angiosome in cases of blindness, suggesting a possible arteriovenous shunting mechanism that could exacerbate such events.^7^ The lack of valves in the venous system in some areas permits retrograde filler flow, increasing the risk of spreading the substance to adjacent and distant anatomical regions, which could amplify complications. In the lip, an arteriovenous anastomosis of capillaries within the vermilion was also identified, heightening the risk of combined vasculature adverse events.^8^

Moreover, the facial venous system has multiple communications with intracerebral veins, which can further compound the severity of adverse events by facilitating the spread of infection from the ‘dangerous triangle’ of the midface to the intracranial venous sinuses.^9^ The lack of valves in these venous pathways underscores the potential for serious complications, including retrograde spread of infections and filler material.

Despite these known risks, there is a notable lack of comprehensive data on the facial vein's course and tributaries, particularly in comparison to those of facial artery.^3^ This review, therefore, aimed to bridge this knowledge gap by providing an illustrated and detailed analysis of the facial venous system. A thorough understanding of the 3D (depth and distribution) anatomy of the facial vasculature is essential for the safe application of non-surgical soft tissue filler procedures.^10^ By expanding the practitioners’ knowledge on venous anatomy, this review seeks to enhance clinical safety and reduce the likelihood of adverse vascular events.

## Methods

The methodology for this narrative review included identifying all published data on the facial vein and its tributaries. Current publications were reviewed to determine the relationship between the venous supply and anatomical danger zones to improve safety in HA dermal fillers. A search strategy was developed and applied to search the Embase (1980–2024) and MEDLINE (1946–2024) databases for relevant literature. A concept table was generated to define the research question according to the patient, intervention, comparator, outcome and study design (PICOS) principle and apply inclusion and exclusion criteria.

### Facial venous vasculature: an overview

**Fig. 1:**
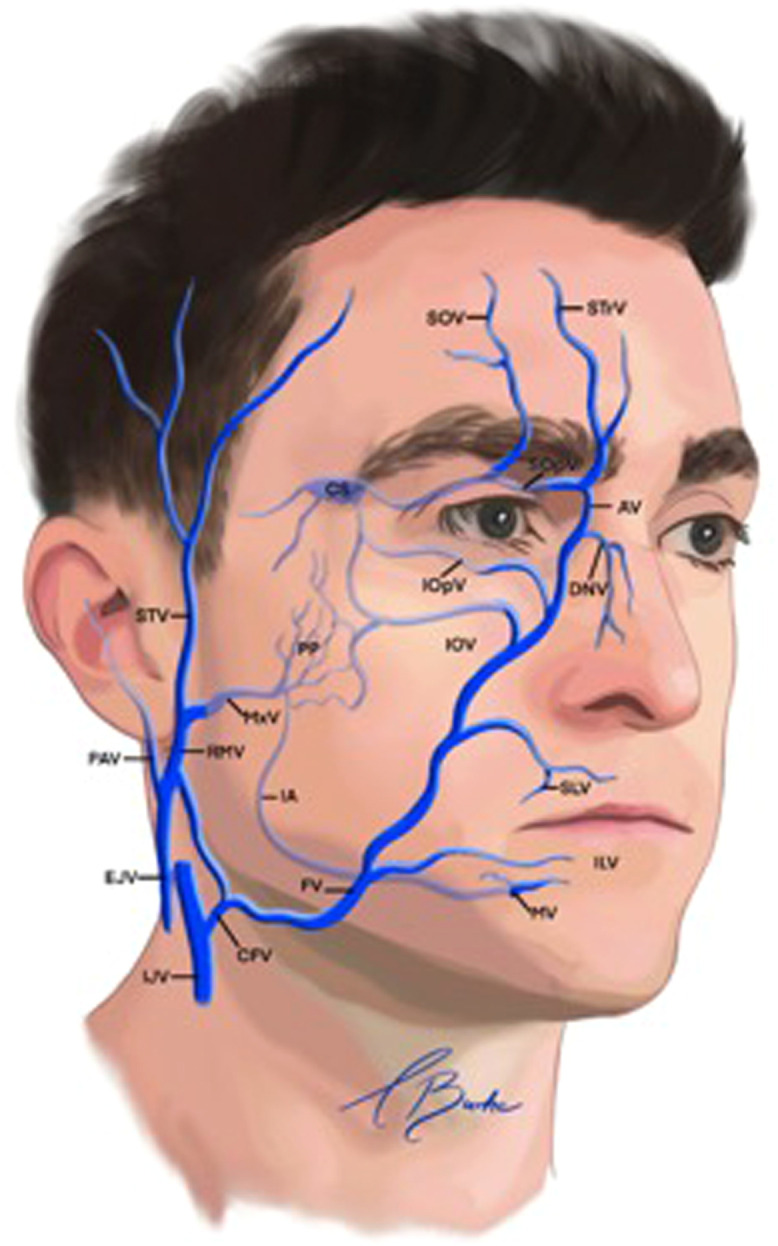
Veins of the face. AV, angular vein; CS, cavernous sinus; EJV, external jugular vein; ENV, external nasal vein; FV, facial vein; IAV, inferior alveolar vein; IJV, internal jugular vein; ILV, inferior labial vein; IOpV, inferior ophthalmic vein; IOV, infraorbital vein; MxV, maxillary vein; MV, mental vein; PAV, posterior auricular vein; PP, pterygoid plexus; RMV, retromandibular vein; SLV, superior labial vein; SOV, supraorbital vein; SOV, superior ophthalmic vein; STV, superficial temporal vein; STrV, supratrochlear vein. Reproduced with permission from von Arx T, Tamura K, Oba Y, Lozanoff S. The face–A vascular perspective. Swiss Dental Journal SSO–Science and Clinical Topics. 2018;128(5):382–92.

The primary cutaneous venous drainage of the facial region is derived from the facial and retromandibular veins (Fig. 1). Although most veins in the face accompany their respective arteries, notable exceptions exist. For instance, the inferior ophthalmic and retromandibular veins do not have corresponding arteries.^9^ Additionally, the venous system exhibits greater variability in distribution patterns compared to the arterial system.^11^

The facial vein can be further categorised into veins in the anterior and posterior divisions. The anterior division has several tributaries, including the angular, lateral and dorsal nasal, inferior palpebral (which drain the lower periorbital region) and superior and inferior labial veins. In contrast, the posterior division comprises tributaries from the superficial temporal and maxillary veins.

Similarly, the retromandibular vein distribution can also be subdivided into anterior and posterior divisions. The veins in the anterior division converge with other veins to form the common facial vein, which subsequently drains into the internal jugular vein.^12^ The posterior division merges with the posterior auricular vein, creating the external jugular vein.^11^^,^^13^

The deep venous vasculature associated with the anterior retromandibular vein includes the maxillary vein, which originates from the infratemporal fossa, and the tributaries from the pterygoid plexus, cavernous sinus, superior and inferior ophthalmic veins, infraorbital vein, inferior alveolar vein and mental vein.

Venous valves are present in the superior ophthalmic vein and its main tributaries, including the supraorbital, angular and facial veins. The orientation of these valve cusps is crucial, as it determines the direction of venous blood flow. A study examining the ophthalmic and facial veins found that blood flow in the facial vein typically drains caudally, whereas the superior ophthalmic vein directs the blood towards the cavernous sinus, and the angular vein drains into either the facial or superior ophthalmic vein.^14^

### Venous supply in the upper third of the face

#### The forehead

The forehead is drained by the supraorbital, supratrochlear and central veins, which ultimately converge into the angular vein.^3^ The supraorbital and supratrochlear veins frequently anastomose with the frontal branch of the superficial temporal vein, which drains the lateral forehead. Additionally, the supraorbital vein forms connections with the sentinel vein in the temporal fossa.^15^ This vein enters the orbit via the supraorbital notch or foramen.

**Fig. 2:**
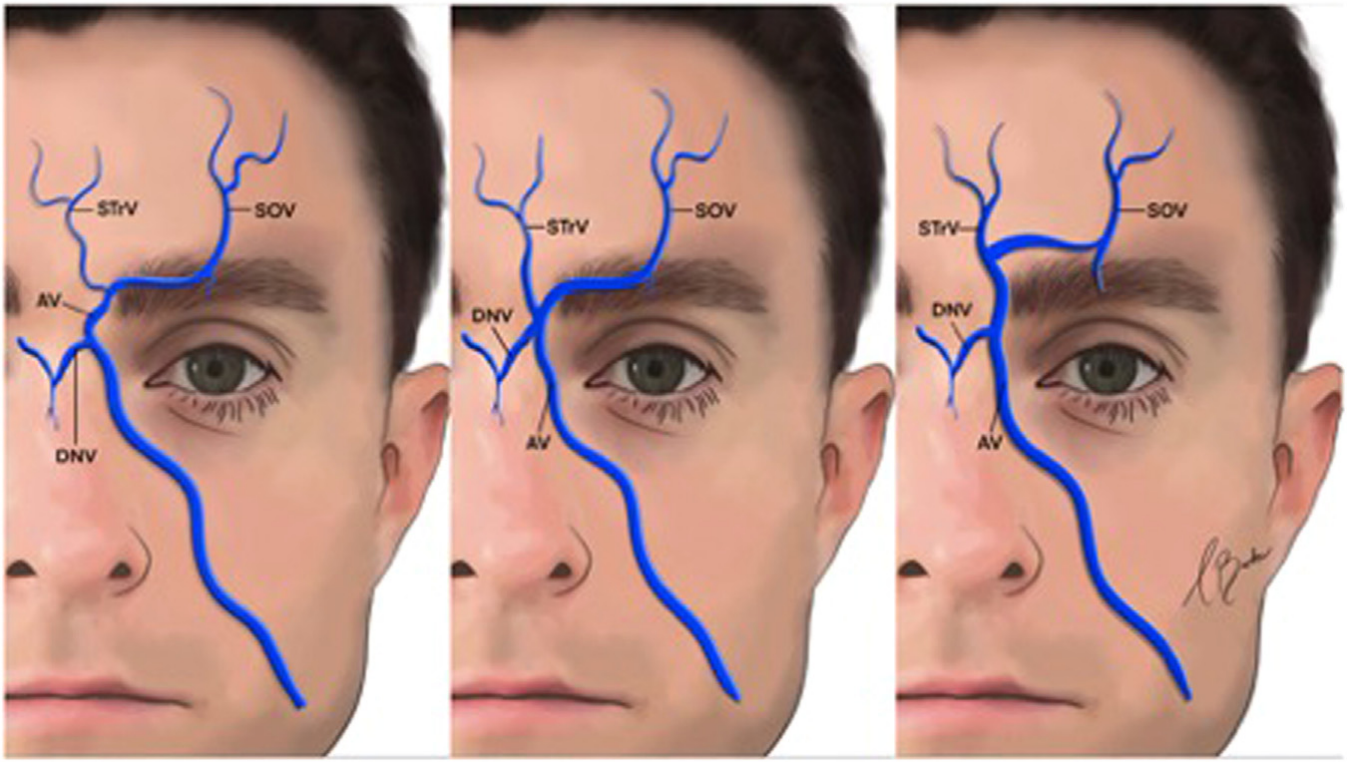
Venous drainage of the forehead. AV, angular vein; DNV, dorsal nasal vein; SOV, supraorbital vein; STrV, supratrochlear vein. Reproduced with permission from Siwetz M, Widni-Pajank H, Hammer N, Pilsl U, Bruneder S, Wree A, Antipova V. The course and variation of the facial vein in the face—Known and unknown facts: An anatomical study. Medicina. 2023;59(8):1479.

The angular vein has a critical connection to the superior ophthalmic vein at the superior orbital margin, facilitating communication with the cavernous sinus. Notably, the central vein often exhibits asymmetry, being larger on one side of the forehead. It has been observed to bifurcate in the inferior third of the forehead, draining into the superomedial orbits.^16^ The central vein typically emerges from the supratrochlear foramen.^15^ Furthermore, three distinct subtypes of the central vein have been proposed,^17^ based on variations in the level and direction of venous drainage (Fig. 2).

#### Temporal fossa

The venous drainage of the temporal fossa is complex and intricate, arising from its superficial, middle and deep layers. The superficial temporal vein has frontal and parietal branches that drain the superficial muscles and skin of the temporal region. It originates from a superficial venous plexus located on the lateral aspect of the head. This vein travels laterally over the zygomatic arch and enters the parotid gland, where it becomes the retromandibular vein after anastomosing with the transverse facial vein.^18^ Notably, the superficial temporal vein exhibits a more varied course and larger supply area compared to the superficial temporal artery.

**Fig. 3:**
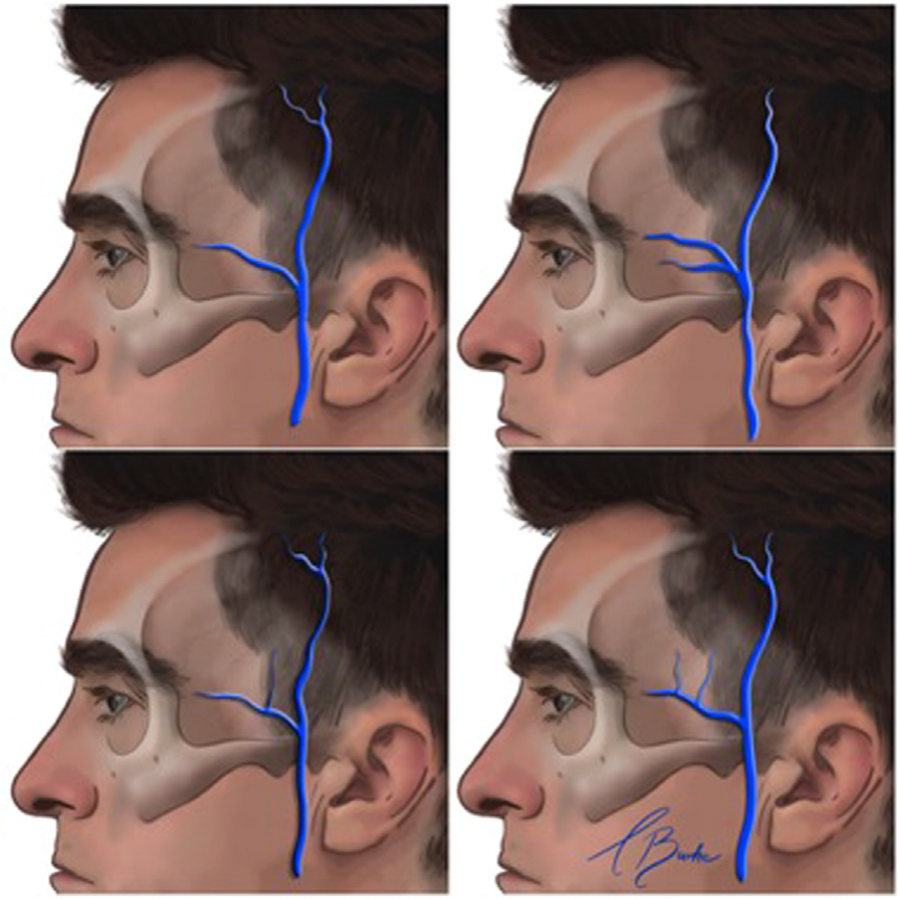
Course and branching pattern of the middle temporal vein (MTV). (Left) Only one major trunk of the MTV is present. (Right) Two major trunks of the MTV. (Lower left) One major trunk of the MTV with a small branch. (Lower right) One major trunk of the MTV with two small branches. Reproduced with permission from Wang D, Xiong S, Zeng N, Wu Y. The middle temporal vein on computed tomographic angiography: Implications for filler injection and reconstructive surgery. Plastic and Reconstructive Surgery. 2023;151(2):315–24.

The superficial temporal vein anastomoses anteroinferiorly with the superior ophthalmic vein via the supraorbital and superior palpebral veins, which are tributaries of the superficial temporal vein.^19^ The middle temporal vein arises from 2 to 4 tributaries at the lateral orbital angle, coursing backwards, downwards and outwards within the temporal fossa (Fig. 3).^20^ It traverses the temporal fossa within the superficial temporal fat compartment, situated between the superficial and deep temporal fasciae, approximately 20 mm superior to the zygomatic arch.^21^

A systematic review of the literature indicated that although the middle temporal vein follows a consistent course and depth, it exhibits significant variability in diameter, ranging from 0.5 mm to 9.1 mm. This vein receives numerous subfascial tributaries from the deeper temporalis muscle. The concept of a ‘venous danger zone’ has been proposed for the inter-fascial planes of the temporal fossa owing to the trajectory of the middle temporal vein within the intermediate fat compartment.^22^ This vein then transitions superficially from the intermediate fat compartment, anastomosing with the superficial temporal vein at the zygomatic arch. Subsequently, the superficial temporal vein descends to form the posterior facial vein after anastomosing with the maxillary vein.

The deep temporal veins run within the temporalis muscle, which is one of the primary muscles of mastication. The posterior auricular vein drains the scalp muscles and skin; it originates behind the ear and lies superficial to the temporal fascia. Following anastomosis superiorly with the occipital vein, the deep cervical veins converge with the posterior branch of the retromandibular vein, which ultimately drains into the external jugular vein.^18^

#### The orbit

**Fig. 4:**
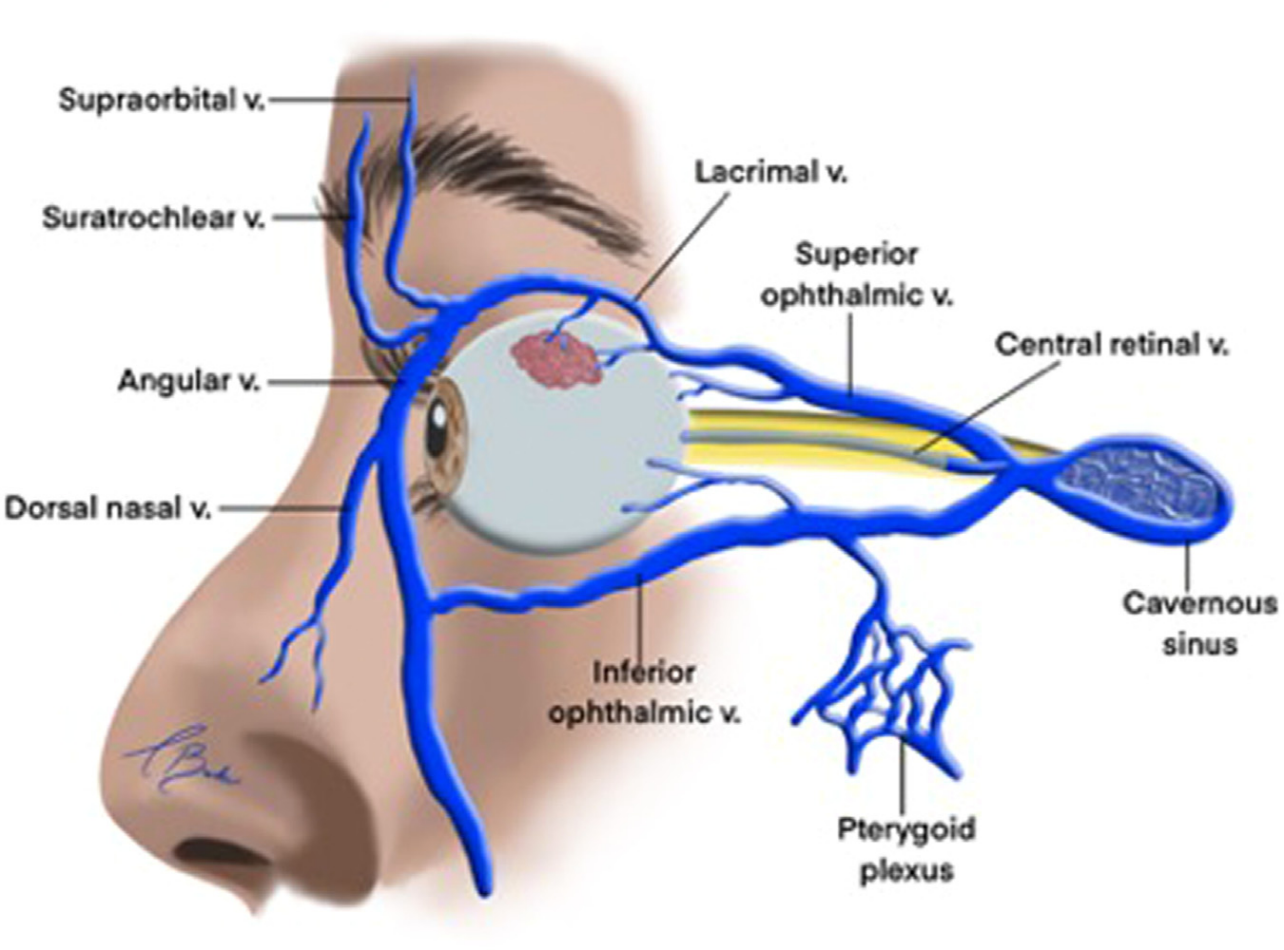
Venous drainage of the orbit. Reproduced with permission from Zhang J, Stringer MD. Ophthalmic and facial veins are not valveless. Clinical & Experimental Ophthalmology. 2010;38(5):502–10.

The bony orbital cavity drains through the ophthalmic veins (Fig. 4). The primary ophthalmic veins are the superior ophthalmic vein (SOV) and inferior ophthalmic vein (IOV), and accessory veins include the medial and middle ophthalmic veins.^23^ Among these, the SOV is the largest. It originates in the anterior medial orbit from the junction of the supraorbital, supratrochlear and angular veins. Within the orbit, the SOV perforates the orbital septum, coursing superiorly and posteriorly through the orbital fat. At the level of the superior orbital rim, the angular vein anastomoses with the SOV. The ophthalmic veins also connect with the facial venous system, draining into the cavernous sinus and intracranial veins.^24^

The IOV arises as a venous plexus located between the globe and anteromedial portion of the orbital floor, positioned above the inferior rectus muscle and below the optic nerve.^14^ The ophthalmic veins serve the essential function of draining blood from the eye and orbit, as well as from the periocular tissues and parts of the midface, including the glabella and nasal bridge. The IOV typically bifurcates into two branches: one exits the orbit via the inferior orbital fissure to drain into the pterygoid venous plexus, while the other exits through the superior orbital fissure, emptying into either the SOV or directly into the cavernous sinus.^21^

Tributaries of the IOV include veins from the inferior oblique, inferior rectus and inferior vortex veins. Blood collected from these tributary vessels usually drains into the internal jugular vein via the cavernous sinus or, less commonly, into the pterygoid plexus, which subsequently drains into either the internal or external jugular vein.^21^

### Venous supply in the middle third of the face

#### The midface

The venous drainage of the midface predominantly occurs through the angular vein, infraorbital vein and pterygoid plexus, all of which connect with the cavernous sinus. The angular vein drains the middle forehead and upper eyelid, linking the supraorbital vein to the ophthalmic veins. It is formed by the union of the supratrochlear and supraorbital veins, descending inferiorly across the nasal margin of the medial palpebral ligament. Notably, the angular vein communicates directly with the SOV. The orbital veins exhibit abundant connections with the extraorbital veins, particularly through the angular veins, which direct anastomoses with the SOV. Consequently, blood can flow from the angular vein inwards to the ophthalmic veins or caudally to the anterior facial vein.

As the angular vein descends alongside the nose, it anastomoses with smaller veins that drain the nose and eyelids. The caudal continuation of the angular vein travels superficially to the levator labii superioris alaeque nasi muscle and deep to the orbicularis oculi, zygomaticus major muscles and buccal fat compartment.^3^ The angular vein serves as an anatomical boundary structure for the fat compartments of the face. It corresponds with the lateral border of the deep medial cheek fat^25^ and premaxillary space (deep nasolabial fat compartment),^3^ as well as the medial border of the deep lateral cheek fat compartments and suborbicularis oculi fat.^25^

In the lower face, the angular or facial vein traverses through the anterior lobe of the buccal fat pad. Thus, the facial vein plays a pivotal role as a topographical landmark structure within the midface. At the junction with the superior labial vein, the angular vein becomes known as the anterior facial vein.^9^ The angular vein courses along areas where facial muscle contractions compress the veins, influencing venous flow because of valve-like mechanisms.^26^

#### The facial vein

The facial vein is subdivided into anterior and posterior facial veins.

#### The anterior facial vein

The facial vein and artery are located in close proximity at the border of the mandible, specifically at the mandibular notch. The facial vein follows a relatively constant course, varying by approximately 7.0%, with a depth variation from the medial canthus to the plane of the mandible.^27^ The anterior facial vein serves as the primary venous drainage pathway for the facial region.^17^ Literature suggests that the anterior facial vein exhibits a predictable course, although variations in distribution and depth are documented.^28^ The facial vein is positioned posterior to the facial artery, anterior to the parotid duct and deep to the zygomaticus major muscle.

The anterior facial vein arises from the angular vein, descending obliquely and laterally towards the anterior portion of the masseter muscle. Deep to the zygomaticus major muscle, the vein branches into the deep and superficial angular veins. The deep branch travels posteriorly, with a mean distance of 27.4 ± 3.0 mm from the inferior orbital margin. The superficial angular vein anastomoses with the palpebral and infraorbital veins as they emerge from the infraorbital foramen.^3^

In its course, the anterior facial vein traverses over the buccinator muscle and runs anterior to the masseter muscle within the facial vein canal. This canal is formed by the anterior portion of the parotid-masseteric fascia of the parotid gland, which creates a fibrous sheath that protects the vein. Notably, this fascia is divided into two laminae that curve around the anterior margin of the masseter muscle, connecting to the buccopharyngeal fascia of the buccinator muscle. The canal encases the vein within fat and loose connective tissue.^3^

As it crosses the border of the mandible, the anterior facial vein courses superficially to the submandibular gland. At the inferior angle of the mandibular gonial region within the parotid gland, this vein anastomoses with the anterior division of the retromandibular vein, ultimately draining into the common facial vein and subsequently into the internal jugular vein.^11^^,^^28^ Tributaries of the facial vein in the anterior facial region include the angular vein, lateral vein of the nose, dorsal vein of the nose, inferior palpebral veins and superior and inferior labial veins.

**Fig. 5:**
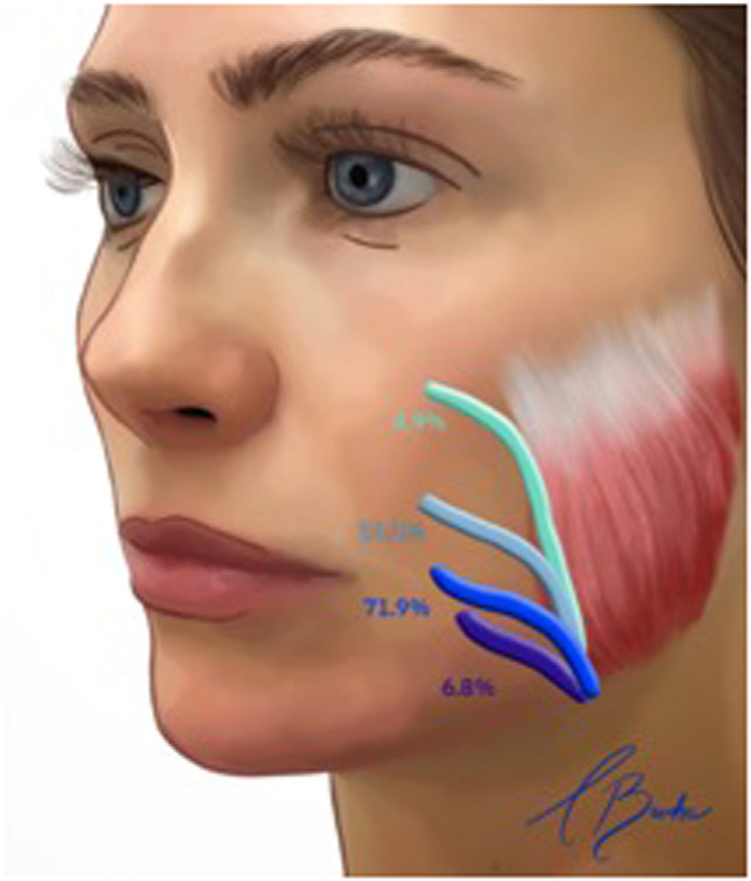
Distribution pattern of the anterior facial vein within the facial vein canal. Reproduced with permission from Siwetz M, Widni-Pajank H, Hammer N, Pilsl U, Bruneder S, Wree A, Antipova V. The course and variation of the facial vein in the face—Known and unknown facts: An anatomical study. Medicina. 2023;59(8):1479.

Variations in the anatomy of the facial vein may diverge from classical anatomical texts (Fig. 1). A recent study identified two types of variation in the course of the anterior facial veins. In type A, 279 facial veins (93.0%) adhered to the standard course, whereas 21 facial veins (7.0%) deviated, crossing the surface of the masseter muscle and parotid duct before running through the parotid gland to join the superficial temporal vein^27^ (Fig. 5).

#### The posterior facial vein

**Fig. 6:**
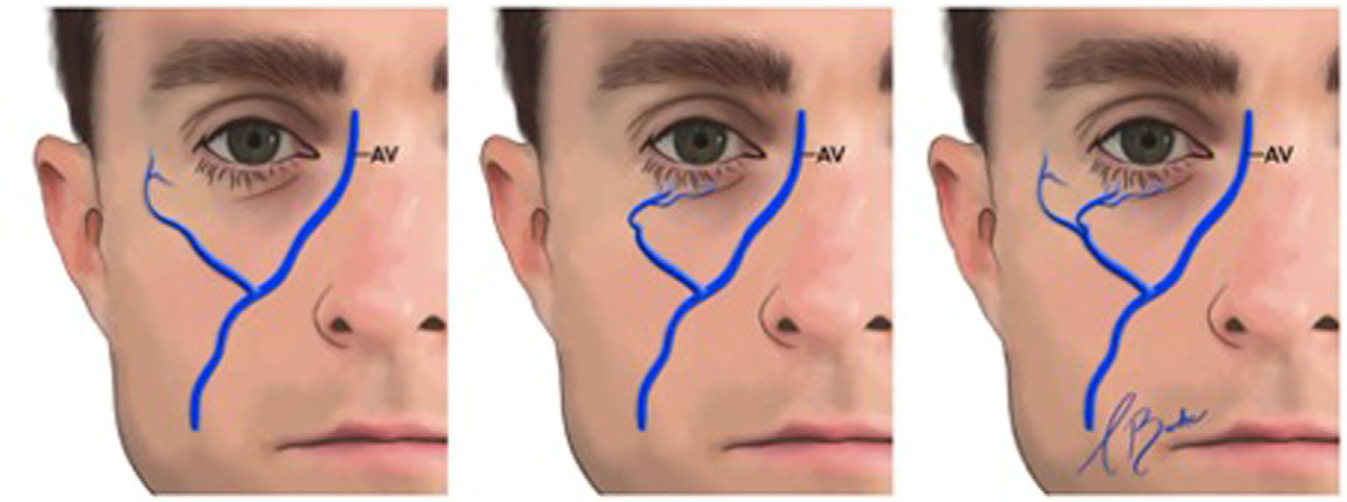
Posterior facial vein. AV, angular vein. Reproduced with permission from Siwetz M, Widni-Pajank H, Hammer N, Pilsl U, Bruneder S, Wree A, Antipova V. The course and variation of the facial vein in the face—Known and unknown facts: An anatomical study. Medicina. 2023;59(8):1479.

Anatomically, the posterior facial vein consists of the union between the maxillary and superficial temporal veins within the parotid gland, resulting in the formation of the posterior retromandibular vein.^11^ The posterior facial vein and its tributaries transition superficially to the buccinator muscle and deep to the masseter muscle.^17^ Both the anterior and posterior facial veins communicate with the pterygoid plexus, receiving input from several source veins. Venous drainage of the midface occurs via the infraorbital vein and the pterygoid plexus, which also connects to the cavernous sinus. The communications between the facial vein and cavernous sinus valves influence the direction of blood flow in the midface, which is significant in the context of infection spread from the face^14^ (Fig. 6).

#### The pterygoid plexus

**Fig. 7:**
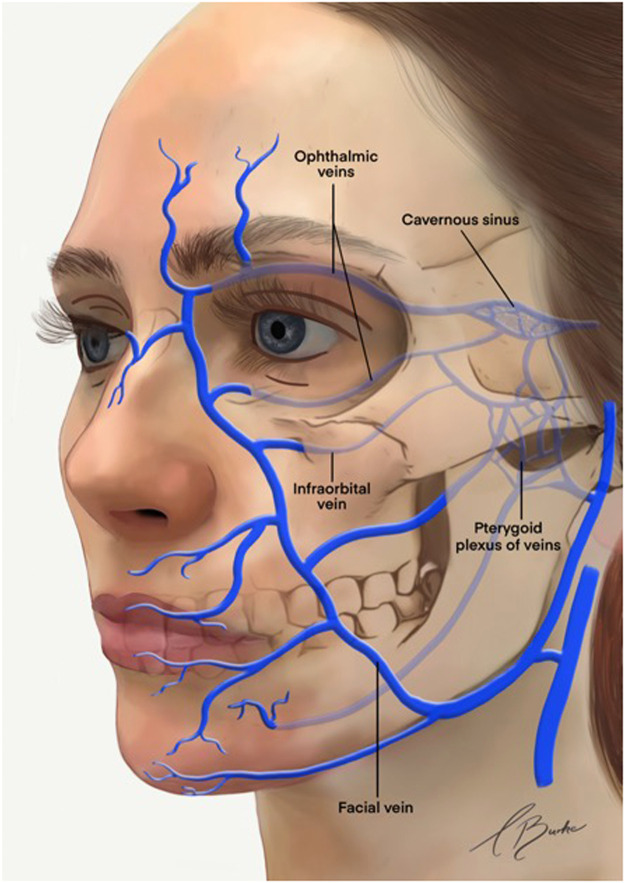
Venous drainage of the cavernous sinus. Reproduced with permission from Tanoue S, Hirohata M, Takeuchi Y, Orito K, Kajiwara S, Abe T. Venous anatomy of the cavernous sinus and relevant veins. Journal of Neuroendovascular Therapy. 2020;14(12):547–57.

The pterygoid plexus is a complex network of veins located within the infratemporal fossa. This plexus communicates extensively with the surrounding veins and anatomical structures.^29^ Positioned between the medial and lateral pterygoid muscles and the temporalis muscle,^30^ the pterygoid plexus also connects with the cavernous sinus, IOV, maxillary vein and facial vein. This communication can potentially allow for the spread of infection through the venous system into the cranium (Fig. 7).

#### The cavernous sinus

The cavernous sinus is a dural venous sinus that drains into the internal jugular vein through the superior and inferior petrosal sinuses and sigmoid sinus. This sinus serves as a vital venous channel, receiving a diverse array of venous structures, which include facial and orbital veins, meningeal veins, as well as superficial and deep veins from the pituitary and cerebral regions.^19^

#### The nose

**Fig. 8:**
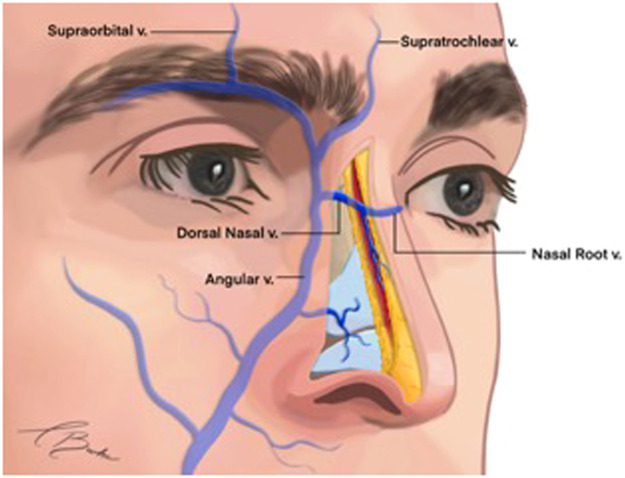
Venous drainage of the nose. Reproduced with permission from Shimizu Y, Imanishi N, Nakajima T, Nakajima H, Aiso S, Kishi K. Venous architecture of the glabellar to the forehead region. Clinical Anatomy, 2013;26(2):183–95.

The nasal veins predominantly follow the course of the nasal arterial supply. The angular vein connects with the dorsal nasal vein, creating an anastomotic venous connection between the right and left hemifaces at the root of the nose,^3^ referred to as the transverse nasal root vein (Fig. 8).^31^ The dorsal vein of the nose extends from the medial angle of the eye along the dorsum towards the root of the nose, where it anastomoses with the angular vein.^17^ These veins can be singular or multiple and typically course laterally within the submuscular layer. The number of lateral nasal veins may vary, ranging from zero to four; interestingly, the external nasal vein is a branch of the angular vein in 100% of cases.^3^ The nasal veins drain into the angular vein located cephalically to the lateral crura and traverse through the upper lateral cartilage, remaining deep to the nasal SMAS and lateral nasal artery. Notably, no large nasal veins are present in the columella.^32^

A significant feature of nasal venous drainage is the direct communication with the cavernous sinus. These veins are valveless, which facilitates the potential spread of infections intracranially.^33^ Notably, this anatomical pathway may allow for the migration of soft tissue fillers into the intracranial circulation.

### Venous supply in the lower third of the face

#### Perioral

**Fig. 9:**
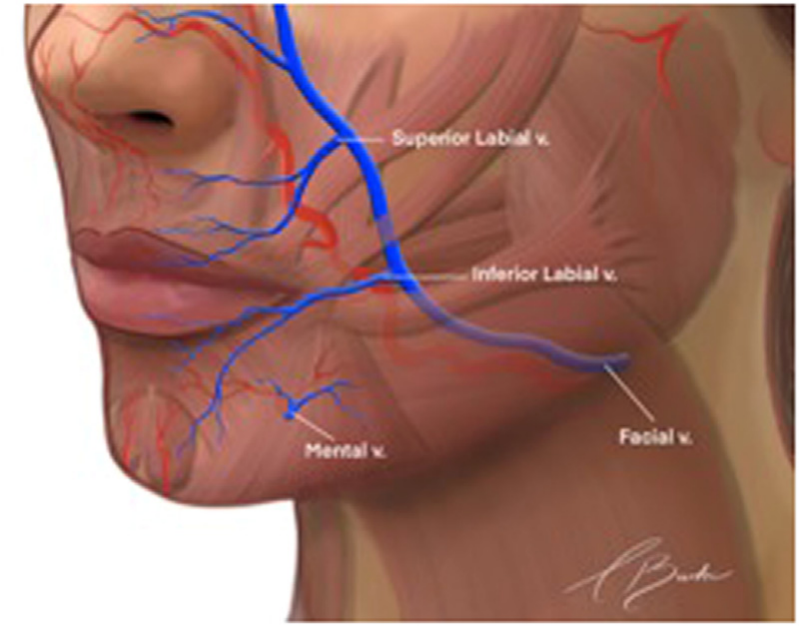
Perioral venous drainage. Reproduced with permission from Moorefield AK, Rose-Reneau Z, Wright BW, Surek CC. Venous tributaries of the lip: Implications for lip filler injection. Plastic and Reconstructive Surgery, 2023;152(2):257e-63e.

The superior and inferior labial veins drain into the anterior facial vein within the facial vein canal (Fig. 9).^3^ These veins are distributed on the cutaneous side of the orbicularis oris muscle.^8^ In fact, a superior labial vein was present in 100% of hemifaces (89/89). This vessel follows a horizontal and lateral course within the upper lip before taking a cranial trajectory; directly above the zygomaticus minor muscle, it anastomoses with the facial vein at the level of the nasal ala in 98% of cases.^17^ The superior labial vein typically has an average fixed course of 15 mm from the superior labial artery.^28^ Anastomoses with the contralateral superior labial vein were identified in only 10% of cases, along with a small number of accessory veins that course parallel to the superior labial vein.^17^ Conversely, the inferior labial vein drains into the facial vein or, in rare instances, forms a common trunk with the superior labial vein. Its drainage point into the facial vein is located at the level of the lower dentition in 84.8% of cases (79/93).^17^ In the lower face, the facial vein traverses through the anterior lobe of the buccal fat pad.^34^ Notably, within the lip, an arteriovenous anastomosis of capillaries has been observed in the vermilion, which could contribute to complications in this aesthetic subunit.^8^

#### The chin

The venous system of the chin drains into the maxillary vein via the mental and inferior alveolar veins. Posterior to the mandibular head, these veins anastomose with the superficial temporal vein, thereby forming the retromandibular vein.^17^ The retromandibular vein comprises two divisions: an anterior deep division and a posterior superficial division. The posterior division merges with the posterior auricular vein to form the external jugular vein,^12^ whereas the anterior division merges with the facial vein, ultimately draining into the internal jugular vein^28^.

## Discussion

Facial vasculature exhibits notable variability in branching patterns (2D) and depth (3D), which has critical implications for procedural safety, particularly in avoiding complications with soft tissue filler injections. Although the arterial system has been a major focus in understanding the causes of these complications, less attention has been paid to the venous system. However, the contribution of the venous system to adverse events is becoming increasingly apparent, especially in the context of intravascular embolisation of soft tissue fillers, which has been documented across all branches of the internal and external carotid arteries.^35^ This reinforces the understanding that no region of the face is entirely free from risk.

Vascular complications, often presenting initially with cutaneous signs, may progress to deeper structures, affecting the subcutaneous fat, muscle, tendons and even bones. If left untreated, the consequences can be severe, leading to ischaemia, tissue necrosis, visual impairment or even more systemic effects such as pulmonary embolisation or stroke.^35^ The relationship between vascular complications and specific anatomical distribution of the arterial and venous networks is critical. For instance, the choke anastomoses of the facial artery define the boundaries of ischaemic risk, whereas true anastomoses enable retrograde flow into the avalvular veins, leading to complications at sites that are distant from the initial injection.^36^

The role of arteriovenous shunts is particularly significant in redirecting large emboli into the venous system, bypassing the capillary bed. This can manifest in territories supplied by the facial or ophthalmic arteries, causing complications far from the injection site. This venous involvement is increasingly recognised, especially given the considerable variation in venous diameters and territories across the face. The complex network of superficial and deep veins, along with their numerous interconnections, adds another layer of risk.

In particular, areas such as the glabella, inner canthi and nasal dorsum present heightened vulnerability to complications owing to their anatomic proximity to the ophthalmic artery and its angiosome. These regions are also more prone to blindness resulting from filler injections, likely due to the potential for arteriovenous shunting in these areas.^37^ Analysing the ophthalmic angiosome and its relationship with other facial vascular territories has highlighted multiple routes for possible arteriovenous shunting, reinforcing the need for careful consideration of the arterial and venous systems in filler procedures.^37^

Although arterial complications tend to manifest rapidly, accidental intravenous injections pose a different risk, with delayed adverse reactions complicating diagnosis and management.^38^ This underlines the importance of expanding research into the venous system's contribution to filler-related complications.

## Conclusions

Understanding the vascular anatomy of the face is essential for ensuring the safe application of aesthetic procedures, particularly those concerning the use of HA fillers. This review highlights the critical role of the facial venous system, which remains under-researched compared to the arterial pathways, despite its significant implications for managing complications such as vascular occlusions and infections. The intricate connections between the facial veins, intracerebral veins and their communication with anatomical danger zones emphasise the need for practitioners to possess a comprehensive understanding of this anatomy. By addressing the knowledge gap regarding the course of facial veins and their tributaries, we aim to enhance clinical safety, reduce the incidence of adverse events and ultimately improve patient outcomes in aesthetic practice.

## Conflicts of interest

None.
